# Context counts: a qualitative study exploring the interplay between context and implementation success

**DOI:** 10.1108/JHOM-07-2020-0296

**Published:** 2021-03-08

**Authors:** Lisa Rogers, Aoife De Brún, Sarah A. Birken, Carmel Davies, Eilish McAuliffe

**Affiliations:** School of Nursing, Midwifery and Health Systems, UCD Centre for Interdisciplinary Research, Education, and Innovation in Health Systems (UCD IRIS), University College Dublin, Dublin, Ireland; UCD Centre for Research, Education and Innovation in Health Systems (UCD IRIS), University College Dublin, Dublin, Ireland; Wake Forest School of Medicine, Winston–Salem, North Carolina, USA

**Keywords:** Teams, Context, Healthcare, Implementation

## Abstract

**Purpose:**

Implementing change in healthcare is difficult to accomplish due to the unpredictability associated with challenging the status quo. Adapting the intervention/practice/program being implemented to better fit the complex context is an important aspect of implementation success. Despite the acknowledged influence of context, the concept continues to receive insufficient attention at the team-level within implementation research. Using two heterogeneous multidisciplinary healthcare teams as implementation case studies, this study evaluates the interplay between context and implementation and highlights the ways in which context influences the introduction of a collective leadership intervention in routine practice.

**Design/methodology/approach:**

The multiple case study design adopted, employed a triangulation of qualitative research methods which involved observation (Case A = 16 h, Case B = 15 h) and interview data (Case A = 13 participants, Case B = 12 participants). Using an inductive approach, an in-depth thematic analysis of the data outlined the relationship between team-level contextual factors and implementation success.

**Findings:**

Themes are presented under the headings: (1) adapting to the everyday realities, a key determinant for implementation success and (2) implementation stimulating change in context. The findings demonstrate a dynamic relationship between context and implementation. The challenges of engaging busy healthcare professionals emphasised that mapping the contextual complexity of a site and adapting implementation accordingly is essential to enhance the likelihood of successful implementation. However, implementation also altered the surrounding context, stimulating changes within both teams.

**Originality/value:**

By exposing the reciprocal relationship between team-level contextual factors and implementation, this research supports the improved design of implementation strategies through better understanding the interplay and mutual evolution of evidence-based healthcare interventions within different contexts.

## Introduction

Healthcare is not static. Change is a “pervasive and persistent” norm for healthcare staff who must continuously respond to technological advancements, changing disease patterns and new treatment discoveries to provide optimal patient care ( Hammer and Champy, 1993 , p. 23; Nilsen *et al.* , 2019 ). However, successful intentional change is difficult to accomplish ( Burnes, 2004 ). The extant literature suggests that two-thirds of change projects fail ( Beer and Nohria, 2000 ; Burnes, 2004 ) and that only 50–60% of care in the past decade aligned with the best available evidence ( Braithwaite, 2018 ). A “one size fits all” approach to implementing change has been associated with this failure, as the priorities of staff and the unique characteristics of settings are overlooked ( Dopson and Fitzgerald, 2005 ; Bauer *et al.* , 2015 ; Braithwaite *et al.* , 2018 ). Therefore, while best practices are intended to apply to all healthcare organisations, accounting for the everyday contextual realities of each setting is necessary to enable the adaptions required to optimise the uptake of change in routine practice ( Berta and Baker, 2004 ).

Although some researchers have previously observed context as nuisance variance to be eliminated, context represents the normal conditions of practice that shape a healthcare team's capacity to implement change ( May *et al.* , 2016 ). Rogers *et al.* (2020a) define context as “…a multi-dimensional construct encompassing micro, meso, and macro level determinants that are pre-existing, dynamic, and emergent throughout the implementation process”. These complex determinants include factors such as culture and leadership which are emerging as influential in the field of implementation science ( Brownson *et al.* , 2012 ). The unpredictability of healthcare settings and the diverse values that exist among team members means that organisations, teams and individuals can respond differently to an intervention (i.e. the prescribed change), and this response usually evolves with time as implementation progresses ( Gadolin and Andersson, 2017 ; Pfadenhauer *et al.* , 2017 ). Therefore, meaningful improvement occurs idiosyncratically and locally ( Braithwaite, 2018 ). Without recognising the variations between contexts (e.g. the norms of those expected to adopt the envisioned change), an implementation effort will likely encounter resistance and subsequently fail ( Braithwaite, 2018 ; Churruca *et al.* , 2019 ). Implementation incorporates strategies and processes. Implementation strategies are cited as influential for improving the uptake of change in routine practice ( Proctor *et al.* , 2013 ). Mazza *et al.* (2013) define implementation strategies as a “purposeful procedure to achieve clinical practice compliance” with an intervention (e.g. incentivising the adoption of the intervention). However, researchers often fail to consider the contextual appropriateness of their predefined strategies and adopt a “kitchen sink” approach (i.e. deploying multiple different strategies) or a standardised “one-size-fits-all” solution ( Harvey and Kitson, 2015 ; Rycroft-Malone, 2015 ). Implementation processes are the complex, multi-level, activities performed to improve the integration of the intervention in practice ( Pfadenhauer *et al.* , 2017 ). These efforts are non-linear and involve multiple actions, refinements, re-evaluations and expansions (e.g. engaging diverse stakeholders and adapting the selected implementation strategy accordingly) ( Damschroder *et al.* , 2009 ). While this study primarily focuses on context and implementation processes, the learning that emerges from this research impacts the development of future implementation strategies. Therefore, all three concepts are used throughout this paper.

While researchers recognise that intervention effectiveness is influenced by its implementation in a specific context, the boundaries between context and implementation are indistinct ( Van Herck *et al.* , 2010 ; Wells *et al.* , 2012 ; Rogers *et al.* , 2020a ). Despite the active influence of context on intervention effectiveness and implementation success, researchers remain primarily concerned with implementation (e.g. the development of implementation strategies), while context continues to receive insufficient attention within implementation research ( Pfadenhauer *et al.* , 2017 ). The interplay between context and implementation processes is unclear with a limited understanding as to how these concepts interact to influence change ( Dryden-Palmer *et al.* , 2020 ). This research responds to these findings by evaluating the interplay between context and implementation success and highlights the ways in which context influences the introduction of change in healthcare practice. While context is a multifaceted concept incorporating multiple levels of the health system, this research focuses primarily on team-level contextual factors as these features have been overlooked in previous implementation research ( Rogers *et al.* , 2020a ). Therefore, context in this research refers to the existing conditions in which healthcare teams operate. However, due to the interdependent relationship between system components, this research also acknowledges the influence of contextual factors at other levels of the health system (i.e. individual, organisational, system) which interact and influence team-level contextual determinants.

## Methods

### Study background

This paper examined the active role of context during the implementation of a team-based collective leadership intervention. From this point forward, throughout the paper this collective leadership intervention will be referred to as “the intervention”. This educational intervention aimed to improve the collective leadership competencies of healthcare teams ( McAuliffe *et al.* , 2017 ). Over a one-year period, four diverse multidisciplinary teams (MDTs) piloted the intervention within the Irish health system. This intervention comprises of eight one-hour team sessions that focus on collective leadership for team performance and safety culture ( Ward *et al.* , 2018 ; Co-Lead, 2019 ). The intervention comprises of six foundational components that are compulsory for each team to complete and an additional 13 targeted interventions which teams can select based on their perceived needs and team priorities. These one-hour team sessions explore topics such as goal setting and role clarity ( Co-Lead, 2019 ). By enabling team members to meet regularly and work through structured sessions that support individual and team learning, the intervention aims to enhance the practice of collective leadership in teams. Appendix 1 provides a reflexive account, detailing researcher characteristics and potential biases.

### Study design

Due to the complexity associated with the concepts of interest (i.e. context and implementation) and the study's aim to generate contextually detailed knowledge relating to implementation, this research adopted a multiple case study design ( Luck *et al.* , 2006 ). A multiple case study describes the assessment of a contemporary phenomenon in two or more real-world settings ( Yin, 2003 ; Stake, 2005 ; Thomas, 2016 ). This approach enabled the researchers to preserve the meaningful characteristics of each team in their real-world environments, supported rich data collection, and allowed the ways in which context and implementation influence one another to be compared across sites ( Yin, 2003 ; Stake, 2006 ; Merriam, 2009 ; Creswell, 2014 ; Harrison *et al.* , 2017 ). In this research, the case study unit was defined as the implementation of the intervention in one MDT. Aligned with Yin's (2003 , p. 85) interpretation of a “good case study”, the study employed a triangulation of qualitative research methods. Employing a variety of data sources enabled converging and contrasting lines of inquiry to be evaluated which increased the trustworthiness of the results ( Lincoln and Guba, 1985 ; Wells, 1987 ; Yin, 2003 ). Observation and interview data assisted with determining “what goes on” in each team, while also eliciting insider descriptions and conceptualisations of the context ( Green and Thorogood, 2014 , p. 157).

### Study sample

To enable an in-depth analysis of context, two of the four multidisciplinary healthcare teams introducing the intervention were chosen as implementation case studies ( Table 1 ). These teams differed in relation to location, size, speciality and governance structure. By applying a context coding framework prior to implementation, distinct cultures were also observed ( Rogers *et al.* , 2020b ). Although one case was described by staff as a traditional hierarchical culture characterised by senior physician control, a more inclusive, multidisciplinary approach was apparent within the other case. Rogers *et al.* (2020) report further information on the culture of each team throughout implementation. Sampling during observational data collection depended on staff availability (i.e. workload and staffing impacted participation). The purposeful sampling strategy adopted during interviews ensured a broad spectrum of perceptions were achieved from a diverse range of disciplines who had varying levels of engagement throughout the implementation process ( Table 2 ). However, the continuous rotation of junior doctors and multi-task attendants (duties include cleaning, portering and catering services on a ward) resulted in their absence from the interview sample.

### Theoretical framework

To gain a greater understanding of implementation, it is essential to monitor contextual factors as the data generated are context dependent. However, despite the reported link between context, implementation and intervention effectiveness, context remains a poorly understood and reported construct within implementation science ( Dopson and Fitzgerald, 2005 ; Nilsen, 2015 ). The Consolidated Framework for Implementation Research (CFIR) is a determinant framework that aids in classifying contextual factors that can influence implementation ( Brownson *et al.* , 2012 ; Tabak *et al.* , 2017 ; Fernandez *et al.* , 2018 ). Determinant frameworks “describe general types of determinants {barriers or facilitators} that are hypothesised or have been found to influence implementation outcomes” ( Nilsen, 2015 ). To develop CFIR, Damschroder *et al.* (2009) consolidated constructs found in 19 published implementation theories, models and frameworks (e.g. Rogers (1995) Diffusion of Innovation Theory, Rycroft-Malone *et al.'s* (2002) Promoting Action on Research Implementation in Health Services framework) and categorised these constructs into five overarching domains: intervention characteristics, outer setting (system-level contextual determinants), inner setting (organisational-level contextual features), individual characteristics (individual-level contextual factors) and the implementation process. Although CFIR identifies 39 constructs that can influence the introduction of change in routine practice, to understand *how* these determinants impact implementation success CFIR in this study is used in conjunction with Proctor *et al.'s* (2011) implementation outcomes. Implementation outcomes are distinct from evidence-based healthcare intervention outcomes and include the concepts of acceptability, adoption, appropriateness, cost, feasibility, fidelity, penetration and sustainability ( Proctor *et al.* , 2011 ). Applying this taxonomy is necessary to understand whether the failure of an intervention is due to an ineffective intervention or whether a potentially effective intervention was deployed incorrectly ( Proctor *et al.* , 2011 ). In this study, the CFIR ( Damschroder *et al.* , 2009 ) and Proctor *et al's* (2011) implementation outcomes guided the design of data collection tools (see below) which assisted with identifying the influence of contextual factors on implementation success. However, this research extends the fields understanding of context. Building on the CFIR and Proctor *et al's* (2011) implementation outcomes, this research enables a deeper evaluation of the impact of team-level contextual factors on implementation success.

### Data collection

#### Observations

Between January and November 2018, 31 hours of non-participant observations were completed at monthly preparation meetings and the intervention sessions (Case A = 16, Case B = 15). Handwritten field notes of phrases quotations and key words relating to participant's conversations, interactions and settings were taken during each observation. Within 24 h of each site visit, these observations were transcribed into detailed accounts to ensure the complete recounting of observed events. These detailed field notes were then inputted into an observation template. This observation template was developed specifically for the needs of this research to evaluate the influence of context on the intervention's implementation ( Appendix 2 ). This customised template incorporates prompts derived from Merriam's (1988) observation checklist. Merriam (1988) provides guidance on the process of collecting observational data during case study research. Due to a dearth of standardised templates currently available for observational research, Merriam (1988) presents a useful checklist of broad factors that are likely to exist in any context. Merriam's (1988) checklist includes questions relating to the setting (physical environment), participants (how many, role descriptions), activities (what is going on) and subtle factors (non-verbal communication, unplanned events). Although these broad factors guided the development of the observation template, to reflect the research aims, these questions were refined and informed by the CFIR ( Damschroder *et al.* , 2009 ) and Proctor *et al.'s* (2011) implementation outcomes. This approach facilitated a structured approach to data collection. For example, question nine within the observation template asks whether participants were actively engaged in the intervention. Field notes relating to staff engagement were inputted into this section of the observation template. The researchers could subsequently understand whether engagement was influenced by Proctor *et al.* 's implementation outcome of acceptability (i.e. did staff satisfaction with the intervention influence their participation?) and/or the CFIR construct of compatibility within the inner setting domain (i.e. did the perceived fit of the intervention with staff values influence staff engagement?).

#### Interviews

Semi-structured interviews were conducted following the implementation of the intervention (February–March 2019). This approach assisted with identifying the dynamic contextual factors influencing implementation from the perspectives of those involved. Theoretical prompts informed by CFIR ( Damschroder *et al.* , 2009 ) and implementation outcomes ( Proctor *et al.* , 2011 ) were included in the topic guide ( Appendix 3 ). In addition to ensuring a consistent analytic framework, the semi-structured interview approach enabled context-sensitive aspects of each case to emerge. One pilot interview was completed which resulted in minimal changes to the structure of the interview schedule. This pilot interview was included in the final data set. In total, 25 MDT members participated ( *n* = 13 Case A, *n* = 12 Case B), and interviews ranged in duration from 18–57 min (average 38 min). All interviews were audio-recorded and transcribed verbatim.

### Data analysis

Thematic analysis, as outlined by Braun and Clarke (2006) , provided the analysis structure. This process involved repeatedly reading data, generating initial codes and developing, refining and naming broader themes. The observational and interview data collected from each team were first coded independently but later converged when developing themes to provide an overall understanding of the interplay between context and implementation within each case of interest. Although the CFIR domains and the implementation outcomes of Proctor *et al.* (2011) informed data collection, the authors chose not to use this as a framework for coding the data, instead favouring an inductive approach to allow for a broader range of codes to emerge from the data. Rather than employing rapid analysis which summarises data into a visual display, this research adopted an in-depth approach that incorporated line-by-line thematic coding ( Gale *et al.* , 2019 ). This method ensured that the developed themes strongly reflected the data collected rather than simply identifying similarities with established CFIR domains and implementation outcomes. NVivo11 software was used to organise the analysis process. Two researchers double-coded the data. This process challenged researcher assumptions, generating new insights and a more complex understanding of the results ( Tracy, 2010 ). One researcher analysed the complete data set, while another double-coded a random 10% of the transcripts. This process enhanced the credibility and dependability of the findings as a high level of agreement was demonstrated in the researchers' coding patterns. Additionally, to enhance transparency a reflexive journal was maintained to map the influence of the researcher throughout the evaluation process (i.e. during observation and interview data collection) ( Lincoln and Guba, 1985 ; Stake, 1995 ; Fossey *et al.* , 2002 ). Reflexive writing ensured the researcher captured what was going on in the field rather than their emotional reaction to, or interpretation of, what occurred during data collection.

### Ethics

Ethics was obtained from the University College Dublin Research Ethics committee (ref: HREC-LS-16–116397) and the participating hospital sites. All participants provided written informed consent during each phase of data collection. To maintain participant anonymity, all potentially identifiable characteristics were removed from each observation and interview transcript. Pseudonyms have also been assigned to both cases to further preserve the anonymity of participants when reporting the results. Willow represents case A, while case B is represented by the pseudonym Brickley ( Table 1 ).

## Results

This study defines implementation success as the extent to which the intervention was completed, routinised and integrated in daily practice. During the eleven-month period of implementation, both teams successfully completed the intervention, implementing the required eight intervention components. Despite national emergencies such as the flu outbreak and extreme weather events which obstructed routine service delivery in 2018, each team recorded adequate attendance throughout implementation (average attendance for both cases = 12 participants). Staff utilisation of the intervention led to the integration of several service improvement initiatives in routine practice ( Table 3 ) and sustained behaviour change within the team (e.g. improved interpersonal relationships).

The key themes emerging from the data demonstrate the nature of the relationship between team-level contextual factors and implementation success. These themes are presented below under the headings (1) adapting to the everyday realities, a key determinant for successful implementation and (2) implementation stimulating change in context. Observation data are represented as Observation *X* , while interview data are presented as the participant's job title (e.g. Nurse1). The cases are distinguished using the letters W for Willow and B for Brickley.

### Adapting to the everyday realities, a key determinant for successful implementation

Despite the differing characteristics of each case ( Table 1 ), the everyday realities impacting implementation were broadly similar. Everyday realities refer to the dynamic contextual characteristics of both cases. The workload of each team, the continuous rotation of its members, varying shift patterns and inadequate staffing were identified as key contextual factors impacting the implementation of the intervention in both cases.

#### Everyday team demands: an active challenge when implementing change

Finding the time to participate in the intervention in addition to staff's busy workload posed a significant challenge for implementing and sustaining the initiative within each MDT. The persistent demands on staff (e.g. high patient turnover, inadequate staffing) impacted the perceived appropriateness of the intervention for some team members. Pressure was a term used by staff to describe the demands of their work and this was exemplified in the observational data. Staff (particularly doctors and allied healthcare professionals (AHPs)) were frequently contacted during each intervention session (Willow = on average 4 bleeps/pager alerts per session, Brickley = on average 1 bleep/pager alert per session). For staff of Willow, the competing demands of the ward required their early departure on occasion (Willow = 6 occurrences during 9 intervention sessions).

Throughout the implementation of the intervention in Brickley, the hospital was in escalation [1] (Observation5B). In the context of this challenging workload, when considering attending the intervention (i.e. team sessions), at times some perceived this commitment as an additional burden. One participant described their thought process when realising an intervention session was planned for that day:

Oh my God, do not tell me I have that to do in the middle of the day along with everything else…the work does not get less (Medic1B)

The staff of Willow considered themselves as “stretched” in their efforts to provide a quality service (Observation14W). One team member suggested that due to their challenging workload, the implementation of the intervention may be perceived as disrupting the workflow of the team, while others suggested that attending the intervention can cause the “pile up of work” (Observation16W). However, in some instances, this demanding context appeared to also enhance the acceptability of the intervention's implementation among teams. In both cases, many staff associated their enjoyment of the intervention to its originality; working on “something that isn't just clinical although it has a clinical kind of effect” (AHP1B). However, the ability of team members to engage with the intervention appeared dependent on the nature of their workload. While they expressed a desire to attend, the unpredictable clinical caseload of frontline staff often limited their presence at the intervention as it was “difficult to just drop everything” (Nurse2B) and “easy to be dragged away” from sessions (Medic1B). However, for those in management positions, commitment to the intervention was suggested as “part of their role” (Nurse4W), which facilitated their continued engagement throughout implementation.

I suppose I'm lucky in my role, I always would have planned the sessions or work around the sessions or put sessions around work (Management2W).

In both sites, inadequate staffing levels impacted the engagement of MDT members. To ensure safe patient care, being “tight on the ground” in relation to staffing (Management1W) implied that teams “could not spare people” to attend (AHP1B). Team members from both sites described their frustration with the insufficiency in staffing which was associated with increased workload, “stress” (AHP1W), and “burnout” (Observation13B). This persistent issue likely reduced the perceived appropriateness of the intervention within each case, potentially impacting the feasibility of implementing the initiative among an under-resourced workforce. Additionally, the fluidity of each team in relation to the rotation of staff and their varying shift patterns limited the potential for widespread understanding and acceptability of the intervention across both MDTs. Nurses and senior physicians were considered the “stable” members of Willow (Nurse1W). However, the participants of Brickley reported that staff rotation was common across all professions. Both teams suggested that the intermittent frequency of the sessions (monthly), the varying shift patterns of some disciplines (e.g. night duty, weekends), and the mobility of team members limited staff engagement with the intervention. As a result, some staff were reported as “not {having} a clue what was going on” (Medic1B). This poor understanding influenced staff investment in the intervention with participants suggesting that some attended simply “due to availability” (Observation12W) while others were obligated; “they had to be there, so they were there” (Nurse4B). Therefore, the mobility of staff within healthcare teams impacts the feasibility of sustaining the intervention, as it requires continuous induction and promotion across the MDT.

#### Supporting change by adapting to local constraints

The influence of these dynamic contextual factors on the intervention's introduction were reduced to some extent by features of the implementation process. Following the initial implementation phase, the timing of the intervention changed to coincide with team members' lunch breaks. Due to the busy context of both sites, some staff disapproved of this decision as they “just want{ed} to have {their} lunch” (AHP3B) and “switch off completely” (Nurse1W). However, for most participants incorporating the intervention into their scheduled break and obtaining a complimentary lunch was considered fundamental for encouraging people to attend. Additionally, the feasibility of implementing the intervention was enhanced due its on-site location. Staff recognised the accessibility of the setting as advantageous in enabling MDT members to leave if they were required on the ward. However, this convenience also facilitated interruptions at both sites (e.g. staff leaving to answer bleeps/pager alerts). Furthermore, due to the dynamics of Brickley ( Table 1 ), the location of the intervention on one ward, impeded the engagement of nursing staff located on the other unit. Staff suggested that nurses are more likely to attend if the initiative is “at a local level” meaning the more visible and proximate the sessions, the more likely staff will attend (Observation14B).

### Implementation stimulating change in context

Although the complex characteristics of each team influenced implementation success, the implementation process also altered the context of each team. In addition to the intervention stimulating change, staff in both sites recognised the *process* of its implementation as influential in enhancing staff camaraderie, role appreciation and job satisfaction within the team.

#### Socially mediated collaboration as a facilitating force

The process of bringing the MDT together, face to face was considered an unusual but beneficial aspect of the implementation process. Irrespective of the intervention content, staff reported valuing the social aspect of implementation which enhanced the soft skills of communication and collegiality within both MDTs. Although the everyday realities of practice impacted the feasibility of attendance by all staff, for those that could engage, the process of giving each discipline a voice helped to break down “fences” between professions (Medic1W). Staff from each case implied that the unique cross-disciplinary approach to implementation heightened familiarity among staff which enabled the establishment of informal relationships.

There was never the platform {to meet as a MDT} before… actually just sitting around and having a cup of tea and a sandwich with somebody is a nice way to actually get to know people…we're not just professional silos that we're people with personalities behind it all (AHP1B).

Bringing the MDT together was predominantly observed positively by participants in both sites. However, for a minority of team members in Willow, the process provoked discomfort. These team members felt “targeted” by other disciplines (AHP3W) due to the deeply ingrained hierarchy evident in this context. To respond to this issue, the intervention was subsequently adapted to include the agreement of ground rules among team members. These ground rules required the removal of professional titles and the use of first names when communicating throughout the intervention's implementation (Observation6W).

#### Team reflexivity: a novel experience

Additionally, rather than “slavishly” completing their routine duties, by implementing the intervention during daily practice, the initiative gave the team a “hiatus” to reflect on how they work as a MDT (Medic1B). Providing a “space to stop” (Observation10W) and strategically plan together was considered a novel experience which provided a “bigger picture” view of team and organisational operations (Nurse2W). This broader perspective re-orientated staff to the importance of collaboration. All participants acknowledged an enhanced appreciation for their colleagues as throughout the process the importance of each role was “validate{d}” (AHP3B). Involving multiple professions and devoting time to understand the challenges each discipline encounters was observed as a “very useful” process (Medic1W). These team reflections were subsequently translated into goals to improve the efficiency of service provision ( Table 3 ).

It just sparks in everyone's memory like why we are all here and working together at the end of the day…what we're all working towards (Nurse4B)

#### Psychological safety: a prerequisite for change

Participants attributed their “frank and open” (AHP1B) discussions to the “relaxed environment” created throughout implementation (Nurse3B). Some staff implied that the provision of refreshments during the intervention's implementation helped establish an informal atmosphere which encouraged team members to “speak freely” (Nurse2W). While several staff considered their familiarity with the location as essential for creating a “comfortable” (Nurse2W), “safe, open” atmosphere (AHP3B). One participant suggested that the neutrality of the location is significant in ensuring the opinions of all staff are respected.

If it's in a neutral setting, you're not discussing a patient {where} somebody, maybe a consultant {senior physician} is in charge, so neutral…everybody's opinion is completely valid (AHP1W)

For a minority of participants in Willow, the confidence to openly discuss their views was dependent on the frequency of their attendance at the intervention. This perception likely reflects the power relations of Willow which perhaps increased staff unease with sharing their opinion during the initial phase of implementation. However, in both cases, when a “safe” (Management2W) atmosphere was created, staff appeared intrinsically motivated to improve practice. Team members reported feeling “able to make a change” (Nurse1B) because “everyone got their chance to have their say” (Support staff1W) and “everyone was treated the same” (AHP4W). Particularly for staff in Willow, this inclusive approach to implementation promoted the value of each team member. Some participants associated this newly recognised worth to enhance job satisfaction.

…if you feel listened to in work…your day is a much nicer day …we are all a chain and no matter how small a link … if that link is broke, it's all broke. So, we should all feel valued (Nurse2W)

## Discussion

Using a multiple case study design and a triangulation of qualitative research methods, this study evaluated the relationship between context and implementation and identified the ways in which team-level contextual factors influence change in healthcare practice. Through investigating an underdeveloped area of study, this research exposed the reciprocal relationship between team-level contextual factors and implementation success, revealing how these concepts dynamically interact, respond and mutually evolve. For example, to successfully integrate the intervention into two teams characterised by numerous competing demands, the implementation process required adaptations at a local level (e.g. timing of sessions tailored to fit each context). However, this influence was reciprocal. Determinants relating to implementation (i.e. delivering the intervention face-to-face, during daily practice, in a familiar, neutral location) enhanced the surrounding context of each team, improving interprofessional communication and relationships. For researchers, policymakers and healthcare professionals (HCPs), the results emphasise the importance of appreciating this bidirectional influence when introducing change to ensure the complex relationships between determinants can be better understood. Equally attending to implementation planning and the context will ensure the selection of appropriate implementation strategies, improving the likelihood of successfully implementing intentional change in routine practice.

The findings underscore the active role of context during an intervention's implementation ( Dryden-Palmer *et al.* , 2020 ). For some HCPs, the everyday demands of their service impacted the perceived appropriateness of implementation. Brownson *et al.* (2012) suggest that successful implementation requires the integration of an initiative into a health setting. If this is not achieved, staff often perceive the intervention's implementation as a time limiting burden ( Geerligs *et al.* , 2018 ). In this study, although attempts were made to reduce the burden of implementation (e.g. onsite location of the intervention), the workload and inadequate staffing of each case hindered the collective engagement of all MDT members. Workload and understaffing were intertwined challenges for frontline staff. The workload of each team member and their limited time for implementation were related to staff shortages. Aligned with the extant literature these dynamic determinants were fundamental barriers to the intervention's adoption ( McAteer *et al.* , 2014 ; Geerligs *et al.* , 2018 ). Staff frustration with their working conditions has been previously documented within the Irish health system ( Health Service Executive, 2018 ). In a national survey, only 50% of HCPs reported satisfaction with their workload, something which resulted in increased stress for MDT members ( Health Service Executive, 2018 ). The impact of workload and inadequate staffing on the psychological well-being of HCPs has been previously outlined ( Gelsema, 2006 ; Hayes *et al.* , 2017 ). The findings from this research, however, reveal the impact of these everyday realities on the introduction of change in routine practice. This study reveals how the feasibility of implementing and sustaining change is dependent on the capacity of teams in an overstretched and under-resourced health system.

The stability in the membership of each team also influenced implementation. Similar to previous literature, the high turnover and varying shift patterns of disciplines obstructed the engagement of some staff ( Geerligs *et al.* , 2018 ). However, the intermittent attendance of these mobile team members may also be advantageous to implementation. The mobility of participants and the knowledge they acquired from the intervention during their rotation on Willow or Brickley may have assisted with the intervention's promotion to other sites across the health system. Therefore, future research should endeavour to follow these transient staff longitudinally to explore whether the mobility of healthcare teams can act as a facilitator to the penetration or spread of knowledge across the wider health service.

Identifying these everyday realities, demonstrates the importance of mapping real-world contexts when introducing change in healthcare practice ( Lau *et al.* , 2016 ; Ellis *et al.* , 2020 ). Observation remains an underused method in implementation research ( Weiner *et al.* , 2011 ); however, the value of this approach is evident in this study. By mapping the landscapes of settings, observations enable change agents to identify and attend to the capacity and readiness of a context when implementing change ( Ellis *et al.* , 2020 ). Recently, methods have been advanced to support researchers, HCPs and policymakers in obtaining a deeper understanding of context ( Rogers *et al.* , 2020b ). By attaining this nuanced perception, interventions and implementation strategies can be adapted to promote their receptivity in diverse contexts. As highlighted in this study, flexibility is required to support implementation (e.g. tailoring the time of the intervention to suit staff workload). Historically, deviation from the research protocol was perceived as a threat to implementation fidelity, compromising the effectiveness of the intervention ( Bopp *et al.* , 2013 ; Chambers and Norton, 2016 ). However, recent literature recognises that although the underlying principles that make the intervention effective require perfect fidelity (core functions), the tailoring of strategies which support each intervention principle (forms) is necessary to improve context fit ( Kirk *et al.* , 2019 ; Perez Jolles *et al.* , 2019 ). Although identifying these core functions and forms strengthen local implementation, these components are rarely specified by intervention developers ( Kirk *et al.* , 2019 ). Future change agents need to disentangle intervention forms from core functions to support the adaptations necessary to implement change in real-world contexts.

The challenges associated with day-to-day practice also enhanced staff enjoyment of implementation. The intervention's implementation was described as a novel opportunity to stop and reflect as an MDT. HCPs are predominantly trained intraprofessionally, in discipline specific groups ( Baxter and Brumfitt, 2008 ). McHugh *et al.* (2020) recently questioned whether team reflexivity is acceptable in varied healthcare environments and requested that future studies acquire a more detailed understanding of how this approach is advantageous in practice. The findings in this study reveal that the unique cross-disciplinary approach heightened staff engagement with the intervention's implementation. Aligned with previous literature rather than performing their routine tasks in isolation, the dedicated time together enabled both MDTs to set priorities and develop quality improvement initiatives to optimise patient care ( Miller *et al.* , 2007 ; Bååthe and Norbäck, 2013 ; Gadolin and Andersson, 2017 ). However, this research also uncovered that the opportunity to collectively reflect heightened camaraderie and staff appreciation across disciplines. Subsequently, these perceived benefits enhanced staff commitment throughout implementation. Although essential activities of implementation have been previously outlined ( Damschroder *et al.* , 2009 ), the originality of an implementation effort has not yet been recognised as an important feature of implementation in the current evidence-base.

Additionally, this study may be of significance to those implementing change as the findings emphasise the importance of socially mediated implementation processes. Previous literature has suggested that social elements of implementation can enhance organisational learning capacity and intervention adoption as the utility of an intervention can be discussed, disputed, and established ( Berta *et al.* , 2015 ; Dryden-Palmer *et al.* , 2020 ). However, the positive impact of the intervention's face-to-face implementation took time to emerge in the context of Willow due to the deeply ingrained hierarchy of this team. Following the adaption of the intervention to include ground rules, the inhibiting effects of status differences between disciplines were weakened. Subsequently, all professions appeared empowered to speak up, began questioning current practice and collaborated in the development of service improvement initiatives. Without this psychological safety, recommending suggestions for change, disregarding professional status boundaries or offering feedback would be too risky for some MDT members ( Nembhard and Edmondson, 2006 ). As shown in this study, when a psychologically safe, inclusive environment is created, team members feel valued by their colleagues, intrinsically motivated to make change which enhances their job satisfaction. Therefore, psychological safety appears to be a prerequisite for socially mediated implementation processes. When a safe environment is established the engagement of MDT members is heightened which can lead to improvements within the surrounding context.

While this research offers new insights, it is important to acknowledge some limitations. To assist the reader in determining the applicability of the findings, thick descriptions of both cases are offered. While the generalisability of the findings remains restricted due to the use of two cases, the potential transferability of the results is increased as the everyday pressures appear broadly similar across health systems. Although the data collection tools employed by this research are not validated, they supported an in-depth evaluation of context which was necessary to achieve the objectives of this research. Additionally, maintaining a reflexive journal throughout the evaluation process enhanced the trustworthiness of the findings ( Lincoln and Guba, 1985 ; Stake, 1995 ; Fossey *et al.* , 2002 ). Reflexive writing helped enhance the credibility and authenticity of the findings by distinguishing participant voices from that of the researchers ( Fossey *et al.* , 2002 ). Due to the transient nature of membership of healthcare teams, the interview data did not represent the experiences of all MDT members (specifically junior doctors and multi-task attendants were not available for interview). However, a diverse sample of HCPs were recruited, and the triangulation of qualitative research methods employed enabled some of these missing views to be represented within the final data set. Participants did not engage in a formal process of checking the accuracy of the findings. While member checking has been recommended as a technique for heightening credibility ( Lincoln and Guba, 1985 ), the approach can also undermine the trustworthiness of results ( Sandelowski, 1993 ). This research emphasises the dynamic nature of context and demonstrates that with the passage of time the meaning and influence of contextual determinants can change. Therefore, the completed observations and interviews may reflect feelings or opinions participants no longer have or may have forgotten. Consequently, formal member checking could have led to the alteration or retraction of important data, restricting the trustworthiness of the findings. Thus, formal member checking was not employed during the evaluation process.

Despite these limitations, this study has practical implications. This study evaluates an underdeveloped area of study and exposes the ways in which context and implementation interact and mutually evolve. By enhancing understanding of this dynamic interplay and emphasising the need for a flexible approach, change agents can more appropriately plan and tailor their efforts to better harmonise the evidence-based practice, implementation process and context. Attending equally to each aspect with respect to the other two will likely reduce stakeholder burden relating to implementation, aid with preparing context receptivity, and subsequently optimise the possibility for successful change in healthcare practice.

## Conclusion

This research employed a multiple case study design and triangulated observation and interview data to evaluate the dynamic relationship between context and implementation and exposed the ways in which these concepts interact, respond and evolve. By outlining the challenges of engaging busy HCPs, the findings demonstrate that mapping the contextual complexity of a site and adapting implementation accordingly is essential to successful implementation. However, implementation was also recognised as altering the surrounding context and stimulating change within both teams. Understanding this reciprocal relationship is fundamental when designing future implementation approaches. Accounting for and attending to these influences will improve the likelihood of translating evidence-based healthcare interventions into routine practice.

## Figures and Tables

**Table 1:** Case characteristics

	Case A (Willow)	Case B (Brickley)
Hospital classification	Hospital can provide 24-h acute surgery, acute medicine and critical care	Hospital provides tertiary and supra-regional care in addition to 24-h acute surgery, acute medicine and critical care
Location	Rural	Urban
Financial and Governance Structure	Statutory hospital–funded and governed by the national government agency, the health service executive (HSE)	Voluntary hospital–acquires greater autonomy as owned by a religious order and subsequently reports to a hospital board rather than the HSE. This hospital type also receives funding from the state
Hospital size	Approximately, 200 bed capacity	Approximately, 600 inpatient bed capacity, 100 day-bed capacity
Team size	*n* = 65	*n* = 73
Team speciality	Surgical	Medical
Team stability	Intern: 3-month rotation Senior house officer: biannual rotation Registrar: biannual/annual rotation Allied health professionals (AHPs): biannual rotation Multi-task attendants: 3-month rotation	Intern: 3-month rotation Senior house officer: biannual rotation Registrar: biannual/annual rotation Junior AHPs:4-6-month rotation
Team location	Doctors and nurses based on one ward, AHPs move across different units in the hospital	Team divided across two wards located on different levels of the hospital. Nursing staff work permanently one of the wards while doctors and AHPs move between units
Team culture	Hierarchical	Collaborative

**Table 2:** Characteristics of interview participants

Case	Participant	Gender	Sessions attended	Sample details
Case A (Willow)	Nurse1W	F	3	Sample included registered nurses and clinical nurse managers
	Nurse2W	F	4	
	Nurse3W	F	2	
	Nurse4W	F	0	
	Management1W	F	8	Sample incorporated senior managers of the organisation
	Management2W	F	8	
	Medic1W	M	5	Sample comprised of senior physicians (consultants and registrars)
	Medic2W	M	5	
	Support Staff1W	M	2	Sample encompassed the views of a healthcare assistant
	AHP1W	F	2	Sample contained various disciplines from the field of allied health
	AHP2W	M	6	
	AHP3W	F	3	
	AHP4W	F	4	
Case B (Brickley)	Nurse1B	F	3	Sample included registered nurses, advanced nurse practitioners and clinical nurse managers
	Nurse2B	F	1	
	Nurse3B	F	2	
	Nurse4B	F	7	
	Nurse5B	M	6	
	Nurse6B	F	4	
	Medic1B	F	7	Sample comprised of senior physicians (consultants and registrars)
	Medic2B	F	4	
	Support Staff1B	M	1	Sample encompassed the views of a healthcare assistant
	AHP1B	F	6	Sample contained various disciplines from the field of allied health
	AHP2B	F	4	
	AHP3B	F	1	

**Table 3:** Implemented change initiatives from each case

Case	Team goal	Implementation action	Exemplar quotes
Willow	Improve the number of delayed discharges	Audit and feedback of rounding times	We mentioned about the doctors' rounds, that was one huge issue…we did a study on the times to see if we could improve things. So I think doctors, they're doing their rounds more efficiently now…which helps patient flow (Nurse3W)
	Improve communication	MDT huddle introduced	the huddle really helps…It's more effective communication at an appropriate time when people can actually focus and take it all on board (AHP4W)
	Improve workflow	Team completed a Lean 5S to organise, clean, develop and sustain a productive work environment	I think things are running a bit, the ward runs smoother (Support staff1W)
Brickley	Improve role clarity	Development of poster to distinguish staff uniforms	{patients} might know who the nurse is from the colour of the uniform, but they would not know who anyone else is (Nurse4B)
	Improve communication	MDT huddle introduced	The MDT huddle is a small thing but it's hugely helpful…just allows us to plan a little bit better for our patients… (Medic1B)
	Improve patient satisfaction	Development of a patient information leaflet to improve awareness of the team	A lot of patients they're not really aware of what the {unit} is or anything so a lot of those things {the leaflet} I thought were really positive to kind of come out of it (AHP2B)

**Figure:**
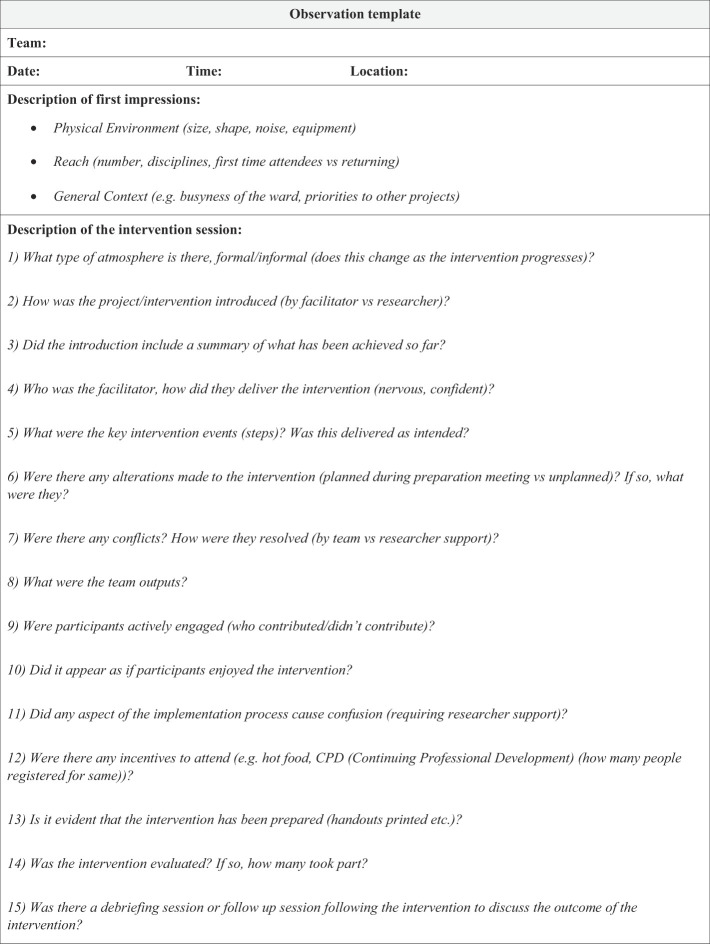


**Table A1:** The association between the observation template questions and Merriam’s (1988) checklist, Proctor *et al.* 's (2011) implementation outcomes and the CFIR Damschroder *et al.* , 2009

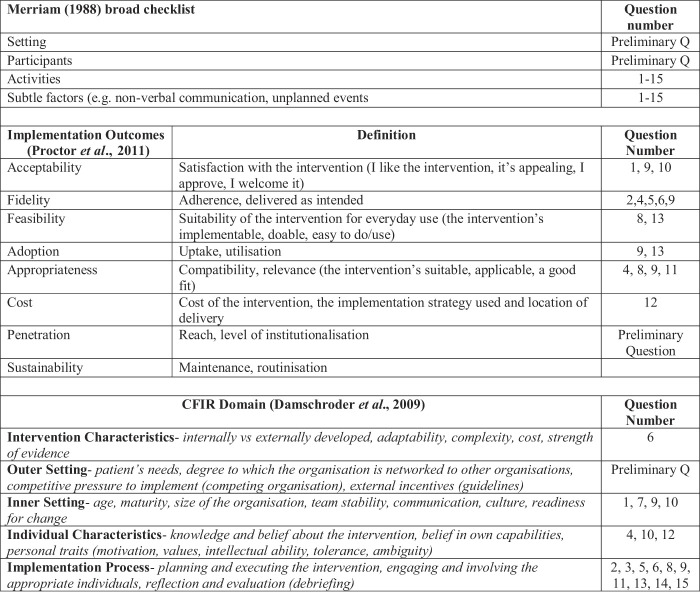

**Table A2:** The association between interview questions and Proctor *et al.* 's (2011) implementation outcomes and the CFIR ( Damschroder *et al.* , 2009 )

Interview question	Implementation outcome	CFIR domain	CFIR constructs
1		Inner setting	Networks and communication Leadership engagement Access to information and knowledge
2	Appropriateness	Intervention characteristics	Relative advantage
		Inner setting	Relative priority
3			
4	Acceptability Feasibility	Inner setting	Culture Leadership engagement Networks and communication Organisational incentives
		Implementation process	Champions
5	Penetration		
6	Acceptability Appropriateness Feasibility	Intervention characteristics	Relative advantage
7	Acceptability Appropriateness Adoption Feasibility Penetration	Intervention characteristics	Relative advantage
		Inner setting	Structural characteristics Available resources
8	Penetration	Intervention characteristics	Relative advantage
		Inner setting	Structural characteristics
9	Penetration	Inner setting	Networks and communication
10	Sustainability		

## Note

1. In escalation refers to an excess demand on emergency and acute services. Escalation protocols consist of a tiered and incremental suite of actions implemented in hospitals to avoid emergency department overcrowding. These escalation procedures seek to ensure the provision of optimal patient care.

## Appendix 1 Researcher reflexivity

The lead author completed each phase of data collection for this research. This researcher is a registered nurse with prior experience working within multidisciplinary healthcare teams. However, the lead author was unfamiliar with the two cases (teams) of study, which heightens their analytical distance from the setting. As qualitative data collection and analysis relies heavily on the researcher's interpretation of events ( Darke *et al.* , 1998 ), the identity of the lead author has influenced this research. However, how or, to what is difficult to predict. This researcher's philosophical approach of post-positivism and epistemological position of pragmatism is derived from their previous experience as a registered nurse. This implies that although the researcher understands the importance of the positivist paradigm of evidence-based practice, they also acknowledge that patient health behaviours are often socially or culturally created, requiring a unique approach to care. Pragmatism is not committed to any one system of philosophy which allowed for the use of different methods, assumptions, and alternative forms of data collection to understand the research problem of interest ( Creswell, 2014 ). The implications of the researcher's epistemological position are evidenced in the theoretical approach adopted by this research. Employing both a determinant framework that aids in classification of contextual factors (i.e. the Consolidated Framework for Implementation Research( Damschroder *et al.* , 2009 )) and a framework of implementation outcomes ( Proctor *et al.* , 2011 ) supported the evaluation of the relationship between context and implementation success. To map researcher influence, a reflexive journal was maintained throughout the evaluation process to enhance transparency of the research ( Lincoln and Guba, 1985 ; Fossey *et al.* , 2002 ). For example, this reflexive journal was updated following each observation session. This allowed the researcher to critically reflect on their initial interpretations of the data without influencing the empirical observational notes. This ensured the researcher captured what was going on in the field rather than their emotional reaction to, or interpretation of, what occurred during the team sessions. To further enhance the trustworthiness of the findings, consultation with the wider research team and regular feedback were additional strategies employed to mitigate researcher impact.

## Appendix 2

Appendix

## Appendix 3 Interview guide

**Aim** : to understand and identify the contextual factors influencing implementation from the perspectives of those involved.

Introduce self, explain research aims briefly, check interviewee has read the information sheet, answer any questions, ask interviewee to sign consent form and advise they can keep copy of information and/or consent sheets. Request permission to record - explain processes.

### Background questions

1. Can you tell me a bit about your professional background / time as a *X* professional?
2. Time in current role / time in current team
3. Can you briefly tell me about your current role and responsibilities in this team (NB the intervention team)?

### Collective leadership intervention implementation

1. How did you hear about the collective leadership intervention?
2. Thinking back to before the collective leadership intervention began can you remember what you (and/or your team) expected from the team sessions?
3. Were you involved in the team sessions in any capacity (as an organiser/facilitator or attendee)? *Probe: There were 8 sessions; can you remember how many you attended?*
4. What influenced your decision or ability to attend (or not attend) the team session(s)?
5. Can you tell me about the typical attendance levels at the sessions?
  - Did the attendance levels change over time? Why?
  - Do you think there was appropriate attendance from all disciplines? Why/why not?
6. What was your impression of the sessions? *What worked well and what did not work so well? Were the sessions relevant to you? Were the sessions enjoyable to attend?*
7. Do you think that the collective leadership intervention worked for your team? *What makes you say this? Why was this the case do you think? How did the sessions lead to that outcome?*
8. Do you think there has been any change in how the team are working or working together as a result of the collective leadership intervention? *What makes you say that? What has changed or what has been initiated through the team's involvement in the collective leadership intervention? What impact have these changes had (on staff, patients, team performance)?*
9. If you're currently working with other teams, have you spread/shared anything you learned through the collective leadership intervention with this team and applied it to your work with other teams or colleagues?
10. Do you think any components of the team sessions that you implemented will be sustained and continued by the team? *Why do you say this? What will the challenges to sustaining this in the team?*

## References

[ref001] Bååthe , F. and Norbäck , L.E. ( 2013 ), “ Engaging physicians in organisational improvement work ”, Journal of Health Organization and Management , Vol. 27 No. 4 , pp. 479 - 497 , doi: 10.1108/JHOM-02-2012-0043 . 24003633

[ref002] Bauer , M.S. , Damschroder , L. , Hagedorn , H. , Smith , J. and Kilbourne , A.M. ( 2015 ), “ An introduction to implementation science for the non-specialist ”, BMC Psychology , Vol. 3 No. 1 , doi: 10.1186/s40359-015-0089-9 . PMC4573926 26376626

[ref003] Baxter , S.K. and Brumfitt , S.M. ( 2008 ), “ Professional differences in interprofessional working ”, Journal of Interprofessional Care , Vol. 22 No. 3 , pp. 239 - 251 , doi: 10.1080/13561820802054655 . 18569411

[ref004] Beer , M. and Nohria , N. ( 2000 ), Breaking the Code of Change , Harvard Business School Press , Boston, MA . 11183975

[ref005] Berta , W.B. and Baker , R. ( 2004 ), “ Factors that impact the transfer and retention of best practices for reducing error in hospitals ”, Health Care Management Review , Vol. 29 No. 2 , pp. 90 - 97 , doi: 10.1097/00004010-200404000-00002 . 15192981

[ref006] Berta , W. , Cranley , L. , Dearing , J.W. , Dogherty , E.J. , Squires , J.E. and Estabrooks , C.A. ( 2015 ), “ Why (we think) facilitation works: insights from organizational learning theory ”, Implementation Science , Vol. 10 No. 1 , p. 141 , doi: 10.1186/s13012-015-0323-0 . 26443999 PMC4596304

[ref007] Bopp , M. , Saunders , R.P. and Lattimore , D. ( 2013 ), “ The tug-of-war: fidelity versus adaptation throughout the health promotion program life cycle ”, Journal of Primary Prevention , Vol. 34 No. 3 , pp. 193 - 207 , doi: 10.1007/s10935-013-0299-y . 23526141

[ref008] Braithwaite , J. ( 2018 ), “ Changing how we think about healthcare improvement ”, British Medical Journal , Vol. 361 , doi: 10.1136/bmj.k2014 . PMC5956926 29773537

[ref009] Braithwaite , J. , Churruca , K. , Long , J.C. , Ellis , L.A. and Herkes , J. ( 2018 ), “ When complexity science meets implementation science: a theoretical and empirical analysis of systems change ”, BioMed Central Medicine , Vol. 16 No. 63 , doi: 10.1186/s12916-018-1057-z . PMC5925847 29706132

[ref010] Braun , V. and Clarke , V. ( 2006 ), “ Using thematic analysis in psychology ”, Qualitative Research in Psychology , Vol. 3 , pp. 77 - 101 , doi: 10.1191/1478088706qp063oa .

[ref011] Brownson , R.C. , Colditz , G.A. and Proctor , E.K. ( 2012 ), Dissemination and Implementation Research in Health: Translating Science to Practice , Oxford University Press , New York, NY, Oxford .

[ref012] Burnes , B. ( 2004 ), “ Emergent change and planned change-competitors or allies? The case of XYZ ”, International Journal of Operations and Production Management , Vol. 24 No. 9 , doi: 10.1108/01443570410552108 .

[ref013] Chambers , D.A. and Norton , W.E. ( 2016 ), “ The adaptome: advancing the science of intervention adaptation ”, American Journal of Preventive Medicine , Vol. 51 4 Suppl 2 , pp. S124 - 131 , doi: 10.1016/j.amepre.2016.05.011 . 27371105 PMC5030159

[ref014] Churruca , K. , Ludlow , K. , Taylor , N. , Long , J.C. , Best , S. and Braithwaite , J. ( 2019 ), “ The time has come: embedded implementation research for health care improvement ”, Journal of Evaluation in Clinical Practice , Vol. 25 No. 3 , pp. 373 - 380 , doi: 10.1111/jep.13100 . 30632246

[ref015] Co-Lead ( 2019 ), “ Collective leadership and safety cultures toolkit, collective leadership and safety cultures ”, available at: https://www.ucd.ie/collectiveleadership/resourcehub/toolkit/ .

[ref016] Creswell , J.W. ( 2014 ), Research Design: Qualitative, Quantitative, and Mixed Methods Approaches , 4th ed. , SAGE , Los Angeles .

[ref017] Damschroder , L.J. , Aron , D.C. , Keith , R.E. , Kirsh , S.R. , Alexander , J.A. and Lowery , J.C. ( 2009 ), “ Fostering implementation of health services research findings into practice: a consolidated framework for advancing implementation science ”, Implementation Science , Vol. 4 No. 50 , doi: 10.1186/1748-5908-4-50 . PMC2736161 19664226

[ref200] Darke , P. , Shanks , G. and Broadbent , M. ( 1998 ), “ Successfully completing case study research: combining rigour, relevance and pragmatism ”, Information Systems Journal , Vol. 8 , pp. 273 - 289 .

[ref018] Dopson , S. and Fitzgerald , L. ( 2005 ), Knowledge to Action?: Evidence-Based Health Care in Context , Oxford University Press , Oxford .

[ref019] Dryden-Palmer , K.D. , Parshuram , C.S. and Berta , W.B. ( 2020 ), “ Context, complexity and process in the implementation of evidence-based innovation: a realist informed review ”, BMC Health Services Research , Vol. 20 No. 1 , p. 81 , doi: 10.1186/s12913-020-4935-y . 32013977 PMC6998254

[ref020] Ellis , J. , Band , R. , Kinsella , K. , Cheetham-Blake , T. , James , E. , Ewings , S. and Rogers , A. ( 2020 ), “ Optimising and profiling pre-implementation contexts to create and implement a public health network intervention for tackling loneliness ”, Implementation Science , Vol. 15 No. 1 , p. 35 , doi: 10.1186/s13012-020-00997-x . 32429961 PMC7238736

[ref021] Fernandez , M.E. , Walker , T.J. , Weiner , B.J. , Calo , W.A. , Liang , S. , Risendal , B. , Friedman , D.B. , Tu , S.P. , Williams , R.S. , Jacobs , S. , Herrmann , A.K. and Kegler , M.C. ( 2018 ), “ Developing measures to assess constructs from the inner setting domain of the consolidated framework for implementation research ”, Implementation Science: IS , Vol. 13 No. 1 , p. 52 , doi: 10.1186/s13012-018-0736-7 . 29587804 PMC5870186

[ref022] Fossey , E. , Harvey , C. , Mcdermott , F. and Davidson , L. ( 2002 ), “ Understanding and evaluating qualitative research ”, Australian and New Zealand Journal of Psychiatry , Vol. 36 No. 6 , doi: 10.1046/j.1440-1614.2002.01100.x . 12406114

[ref023] Gadolin , C. and Andersson , T. ( 2017 ), “ Healthcare quality improvement work: a professional employee perspective ”, International Journal of Health Care Quality Assurance , Vol. 30 No. 5 , pp. 410 - 423 , doi: 10.1108/IJHCQA-02-2016-0013 . 28574326

[ref024] Gale , R.C. , Wu , J. , Erhardt , T. , Bounthavong , M. , Reardon , C.M. , Damschroder , L.J. and Midboe , A.M. ( 2019 ), “ Comparison of rapid vs in-depth qualitative analytic methods from a process evaluation of academic detailing in the Veterans Health Administration ”, Implementation Science , Vol. 14 No. 1 , p. 11 , doi: 10.1186/s13012-019-0853-y . 30709368 PMC6359833

[ref025] Geerligs , L. , Rankin , N.M. , Shepherd , H.L. and Butow , P. ( 2018 ), “ Hospital-based interventions: a systematic review of staff-reported barriers and facilitators to implementation processes ”, Implementation Science , Vol. 13 No. 1 , doi: 10.1186/s13012-018-0726-9 . PMC5824580 29475440

[ref026] Gelsema , T. ( 2006 ), “ A longitudinal study of job stress in the nursing profession: causes and consequences ”, Journal of Nursing Management , Vol. 14 No. 4 , pp. 289 - 299 , doi: 10.1111/j.1365-2934.2006.00635.x . 16629843

[ref027] Green , J. and Thorogood , N. ( 2014 ), Qualitative Methods for Health Research , 3rd ed. , SAGE , London, Los Angeles .

[ref028] Hammer , M. and Champy , J. ( 1993 ), Re‐engineering the Corporation , Nicholas Brealey , London .

[ref029] Harrison , H. , Birks , M. , Franklin , R. and Mills , J. ( 2017 ), “ Case study research: foundations and methodological orientations ”, Forum Qualitative Sozialforschung / Forum for Qualitative Social Research , Vol. 18 No. 1 , doi: 10.17169/fqs-18.1.2655 .

[ref030] Harvey , G. and Kitson , A. ( 2015 ), “ Translating evidence into healthcare policy and practice: single versus multi-faceted implementation strategies – is there a simple answer to a complex question? ”, International Journal of Health Policy and Management , Vol. 4 No. 3 , pp. 123 - 126 , doi: 10.15171/ijhpm.2015.54 . 25774368 PMC4357977

[ref031] Hayes , B. , Prihodova , L. , Walsh , G. , Doyle , F. and Doherty , S. ( 2017 ), “ What's up doc? A national cross-sectional study of psychological wellbeing of hospital doctors in Ireland ”, BMJ Open , Vol. 7 No. 10 , p. e018023 , doi: 10.1136/bmjopen-2017-018023 . PMC5652523 29042389

[ref032] Health Service Executive ( 2018 ), Your Opinion Counts: Results of Health Sector National Staff Survey 2018 , Ipsos MRBI , available at: https://www.hse.ie/eng/staff/staffsurvey/ ( accessed 3 November 2020 ).

[ref033] Kirk , M.A. , Haines , E.R. , Rokoske , F.S. , Powell , B.J. , Weinberger , M. , Hanson , L.C. and Birken , S.A. ( 2019 ), “ A case study of a theory-based method for identifying and reporting core functions and forms of evidence-based interventions ”, Translational Behavioral Medicine , Vol. 11 No. 1 , doi: 10.1093/tbm/ibz178 . PMC7877297 31793635

[ref034] Lau , E.Y. , Saunders , R.P. and Pate , R.R. ( 2016 ), “ Factors influencing implementation of a physical activity intervention in residential children's homes ”, Prevention Science: The Official Journal of the Society for Prevention Research , Vol. 17 No. 8 , pp. 1002 - 1011 , doi: 10.1007/s11121-016-0692-x . 27539092

[ref035] Lincoln , Y.S. and Guba , E.G. ( 1985 ), Naturalistic Inquiry , SAGE , Beverly Hill, CA .

[ref036] Luck , L. , Jackson , D. and Usher , K. ( 2006 ), “ Case study: a bridge across the paradigm ”, Nursing Inquiry , Vol. 13 No. 2 , pp. 103 - 109 , doi: 10.1111/j.1440-1800.2006.00309.x . 16700753

[ref037] May , C.R. , Johnson , M. and Finch , T. ( 2016 ), “ Implementation, context and complexity ”, Implementation Science , Vol. 11 No. 141 , doi: 10.1186/s13012-016-0506-3 . PMC5069794 27756414

[ref038] Mazza , D. , Bairstow , P. , Buchan , H. , Chakraborty , S.P. , Van Hecke , O. , Grech , C. and Kunnamo , I. ( 2013 ), “ Refining a taxonomy for guideline implementation: results of an exercise in abstract classification ”, Implementation Science , Vol. 8 No. 1 , p. 32 , doi: 10.1186/1748-5908-8-32 . 23497520 PMC3606141

[ref039] McAteer , J. , Stone , S. , Fuller , C. and Michie , S. ( 2014 ), “ Using psychological theory to understand the challenges facing staff delivering a ward-led intervention to increase hand hygiene behavior: a qualitative study | elsevier enhanced reader ”, American Journal of Infection Control , Vol. 42 , pp. 495 - 499 , doi: 10.1016/j.ajic.2013.12.022 . 24656784

[ref040] McAuliffe , E. , De Brún , A. , Ward , M. , O’Shea , M. , Cunningham , U. , O’Donovan , R. , McGinley , S. , Fitzsimons , J. , Corrigan , S. and McDonald , N. ( 2017 ), “ Collective leadership and safety cultures (Co-Lead): protocol for a mixed-methods pilot evaluation of the impact of a co-designed collective leadership intervention on team performance and safety culture in a hospital group in Ireland ”, BMJ Open , Vol. 7 , doi: 10.1136/bmjopen-2017-017569 . PMC5695301 29101137

[ref041] McHugh , S.K. , Lawton , R. , O’Hara , J.K. and Sheard , L. ( 2020 ), “ Does team reflexivity impact teamwork and communication in interprofessional hospital-based healthcare teams? A systematic review and narrative synthesis ”, BMJ Quality and Safety , Vol. 29 No. 8 , doi: 10.1136/bmjqs-2019-009921 . PMC7398296 31911544

[ref042] Merriam , S.B. ( 1988 ), Case Study Research in Education: A Qualitative Approach , Jossey-Bass , San Francisco; CA .

[ref043] Merriam , S.B. ( 2009 ), Qualitative Research: A Guide to Design and Implementation , 2nd ed. , SAGE , Thousand Oaks, CA .

[ref044] Miller , K. , Walmsley , J. and Williams , S. ( 2007 ), “ Shared leadership: an idea whose time has come in healthcare? ”, The International Journal of Leadership in Public Services; Hove , Vol. 3 No. 4 , pp. 24 - 37 , doi: 10.1108/17479886200700027 .

[ref045] Nembhard , I.M. and Edmondson , A.C. ( 2006 ), “ Making it safe: the effects of leader inclusiveness and professional status on psychological safety and improvement efforts in health care teams ”, Journal of Organizational Behavior , Vol. 27 No. 7 , pp. 941 - 966 , doi: 10.1002/job.413 .

[ref046] Nilsen , P. ( 2015 ), “ Making sense of implementation theories, models and frameworks ”, Implementation Science , Vol. 10 No. 1 , p. 53 , doi: 10.1186/s13012-015-0242-0 . 25895742 PMC4406164

[ref047] Nilsen , P. , Schildmeijer , K. , Ericsson , C. , Seing , I. and Birken , S. ( 2019 ), “ Implementation of change in health care in Sweden: a qualitative study of professionals' change responses ”, Implementation Science , Vol. 14 No. 1 , p. 51 , doi: 10.1186/s13012-019-0902-6 . 31088483 PMC6518624

[ref048] Perez Jolles , M. , Lengnick-Hall , R. and Mittman , B.S. ( 2019 ), “ Core functions and forms of complex health interventions: a patient-centered medical home illustration ”, Journal of General Internal Medicine , Vol. 34 No. 6 , pp. 1032 - 1038 , doi: 10.1007/s11606-018-4818-7 . 30623387 PMC6544719

[ref049] Pfadenhauer , L.M. , Gerhardus , A. , Mozygemba , K. , Lysdahl , K.B. , Booth , A. , Hofmann , B. , Wahlster , P. , Polus , S. , Burns , J. , Brereton , L. and Rehfuess , E. ( 2017 ), “ Making sense of complexity in context and implementation: the Context and Implementation of Complex Interventions (CICI) framework ”, Implementation Science , Vol. 12 No. 1 , doi: 10.1186/s13012-017-0552-5 . PMC5312531 28202031

[ref051] Proctor , E.K. , Powell , B.J. and McMillen , J.C. ( 2013 ), “ Implementation strategies: recommendations for specifying and reporting ”, Implementation Science , Vol. 8 No. 1 , doi: 10.1186/1748-5908-8-139 . PMC3882890 24289295

[ref050] Proctor , E. , Silmere , H. , Raghavan , R. , Hovmand , P. , Aarons , G. , Bunger , A. , Griffey , R. and Hensley , M. ( 2011 ), “ Outcomes for implementation research: conceptual distinctions, measurement challenges and research agenda ”, Administration and Policy in Mental Health , Vol. 38 No. 2 , pp. 65 - 76 , doi: 10.1007/s10488-010-0319-7 . 20957426 PMC3068522

[ref052] Rogers , E.M. ( 1995 ), Diffusion of Innovations , Free Press , New York, NY .

[ref053] Rogers , L. , De Brún , A. , Birken , S.A. , Davies , C. and McAuliffe , E. ( 2020 ), “ The micropolitics of implementation; a qualitative study exploring the impact of power, authority, and influence when implementing change in healthcare teams ”, BMC Health Services Research , Vol. 20 No. 1059 , doi: 10.1186/s12913-020-05905-z . PMC7684932 33228702

[ref054] Rogers , L. , De Brún , A. and McAuliffe , E. ( 2020a ), “ Defining and assessing context in healthcare implementation studies: a systematic review ”, BMC Health Services Research , Vol. 20 No. 591 , doi: 10.1186/s12913-020-05212-7 . PMC7322847 32600396

[ref055] Rogers , L. , De Brún , A. and McAuliffe , E. ( 2020b ), “ Development of an integrative coding framework for evaluating context within implementation science ”, BMC Medical Research Methodology , Vol. 20 No. 158 , doi: 10.1186/s12874-020-01044-5 . PMC7296653 32539710

[ref056] Rycroft-Malone , J. ( 2015 ), “ It's more complicated than that ”, International Journal of Health Policy and Management , Vol. 4 No. 7 , pp. 481 - 482 , doi: 10.15171/ijhpm.2015.67 . 26188813 PMC4493589

[ref057] Rycroft-Malone , J. , Harvey , G. , Kitson , A. , McCormack , B. , Seers , K. and Titchen , A. ( 2002 ), “ Getting evidence into practice: ingredients for change ”, Nursing Standard , Vol. 16 No. 37 , pp. 38 - 43 , doi: 10.7748/ns2002.05.16.37.38.c3201 . 12068568

[ref058] Sandelowski , M. ( 1993 ), “ Rigor or Rigor Mortis: the problem of rigor in qualitative research revisited ”, Advances in Nursing Practice , Vol. 16 No. 2 , pp. 1 - 8 , doi: 10.1097/00012272-199312000-00002 . 8311428

[ref059] Stake , R.E. ( 1995 ), The Art of Case Study Research , SAGE , CA, London .

[ref060] Stake , R.E. ( 2005 ), “ Qualitative case studies ”, in The SAGE Handbook of Qualitative Research , 3rd ed. , SAGE , Thousand Oaks, CA .

[ref061] Stake , R.E. ( 2006 ), Multiple Case Study Analysis , Guilford , New York, NY .

[ref062] Tabak , R.G. , Padek , M.M. , Kerner , J.F. , Stange , K.C. , Proctor , E.K. , Dobbins , M.J. , Colditz , G.A. , Chambers , D.A. and Brownson , R.C. ( 2017 ), “ Dissemination and implementation science training needs: insights from practitioners and researchers ”, American Journal of Preventive Medicine , Vol. 52 No. 3S3 , pp. S322 - S329 , doi: 10.1016/j.amepre.2016.10.005 . 28215389 PMC5321656

[ref063] Thomas , G. ( 2016 ), How to Do Your Case Study , 2nd ed. , SAGE , London, Los Angeles .

[ref064] Tracy , S.J. ( 2010 ), “ Qualitative quality: eight ‘big-tent’ criteria for excellent qualitative research ”, Qualitative Inquiry , Vol. 16 No. 10 , pp. 837 - 851 , doi: 10.1177/1077800410383121 .

[ref065] Van Herck , P. , Vanhaecht , K. , Deneckere , S. , Bellemans , J. , Panella , M. , Barbieri , A. and Sermeus , W. ( 2010 ), “ Key interventions and outcomes in joint arthroplasty clinical pathways: a systematic review ”, Journal of Evaluation in Clinical Practice , Vol. 16 , pp. 39 - 49 , doi: 10.1111/j.1365-2753.2008.01111.x . 20367814

[ref066] Ward , M.E. , Brún , A.D. , Beirne , D. , Conway , C. , Cunningham , U. , English , A. , Fitzsimons , J. , Furlong , E. , Kane , Y. , Kelly , A. , McDonnell , S. , McGinley , S. , Monaghan , B. , Myler , A. , Nolan , E. , O’Donovan , R. , O’Shea , M. , Shuhaiber , A. and McAuliffe , E. ( 2018 ), “ Using co-design to develop a collective leadership intervention for healthcare teams to improve safety culture ”, International Journal of Environmental Research and Public Health , Vol. 15 , pp. 1 - 17 , doi: 10.3390/ijerph15061182 . PMC6025638 29874883

[ref067] Weiner , B.J. , Amick , H.R. , Lund , J.L. , Lee , S.D. and Hoff , T.J. ( 2011 ), “ Review: use of qualitative methods in published health services and management research: a 10-year review ”, Medical Care Research and Review , Vol. 68 No. 1 , pp. 3 - 33 , doi: 10.1177/1077558710372810 . 20675353 PMC3102584

[ref068] Wells , K. ( 1987 ), “ Scientific issues in the conduct of case studies ”, Journal of Child Psychology and Psychiatry , Vol. 28 , pp. 783 - 790 , doi: 10.1111/j.1469-7610.1987.tb00668.x . 3325519

[ref069] Wells , M. , Williams , B. , Treweek , S. , Coyle , J. and Taylor , J. ( 2012 ), “ Intervention description is not enough: evidence from an in-depth multiple case study on the untold role and impact of context in randomised controlled trials of seven complex interventions ”, Trials , Vol. 13 No. 95 , doi: 10.1186/1745-6215-13-95 . PMC3475073 22742939

[ref070] Yin , R.K. ( 2003 ), Case Study Research: Design and Methods , 3rd ed. , SAGE , Thousand Oaks, CA, London .

